# Leisure-time physical activity among adolescents and subsequent use of antidepressant and hypnotic drugs: a prospective register linkage study

**DOI:** 10.1007/s00787-018-1160-x

**Published:** 2018-05-02

**Authors:** Annette Løvheim Kleppang, Ingeborg Hartz, Miranda Thurston, Curt Hagquist

**Affiliations:** 1 Department of Public Health, Inland Norway University of Applied Sciences, Campus Elverum, Terningen Arena, PO Box 400, 2418 Elverum, Norway; 2 Centre for Research on Child and Adolescent Mental Health, Karlstad University, Karlstad, Sweden; 3 Division of Epidemiology, Norwegian Institute of Public Health, Oslo, Norway; 4 Inland Hospital Trust, Harstad, Norway

**Keywords:** Adolescents, Leisure time, Physical activity, Psychotropic drug use, Prescription database, Longitudinal study

## Abstract

In this prospective study, the association between physical activity and subsequent use of antidepressant and hypnotic drug use in adolescents aged 15–16 years was examined. This study is based on information retrieved from the Norwegian Youth Health Surveys (2000–2003) and linked to prescription data from the Norwegian Prescription Database (2004–2013). In total, the study included 10711 participants with a participation rate of 87%. Adolescents were asked how many hours per week they spent on physical activity that made them sweat and/or be out of breath outside of school. Incident psychotropic drug use (outcome measure) was defined as ≥ 1 prescription of one of the following psychotropic drugs: hypnotics and antidepressants registered in the Norwegian Prescription Database. In the crude model for the time period 2004–2007, the odds of incident hypnotic use were lower for those who were physically active 1–2 h per week (OR 0.48–0.64), compared to those who were physically inactive (< 1 h per week). However, the association become non-significant 4-year post-baseline (2008–2010 and 2011–2013). In the crude model for the time periods 2004–2007, 2008–2010 and 2011–2013, the odds of incident antidepressant use were lower for physically active adolescents (2004–2007: OR 0.46–0.71, 2008–2010: OR 0.40–0.67 and 2011–2013: OR 0.37–0.58, compared to those who were physically inactive < 1 h. However, after adjustment for confounders, the association became non-significant in all time periods except in physical activity 5–7 and 8–10 h in the period 2008–2010. Physical activity does not indicate any association with later use of antidepressants, and the significant association with incident hypnotic drug use was for short-term follow-up only and disappeared on longer term follow-up periods. Given the scarcity of longitudinal studies examining the association between physical activity and mental health as well as psychotropic drug use among young people, the current study adds to previous research.

## Introduction

The prevalence of mental health difficulties among adolescents has increased in recent years with problems having an earlier age of onset (between 12 and 24 years of age) [1]. Alongside these trends, the use of antidepressants has also increased markedly in many countries. In Norway, for example, antidepressant use has increased among adolescents (16–17 years) over the past few years, especially among girls [2]. Similarly, there has been an increase in hypnotic drug use among Norwegian youth in the period 2006–2010 [3, 4]. It is also noteworthy that ‘many psychiatric disorders with an adolescent onset have a strong continuity with adult disorders and, therefore, contribute a sizeable proportion of adult psychiatric morbidity’ [5]. From a public health perspective, identifying modifiable factors that strengthen young people’s mental health has the potential to reduce the risk of future mental illness and the subsequent use of psychotropic drugs. This paper focuses on the putative role of physical activity as a modifiable protective factor.

In recent years, concerns have been expressed relating to the decline in physical activity alongside a rise in sedentary behaviour during adolescence [6]. This is important given that higher levels of sedentary behaviour have been associated with worse mental health [7]. Higher levels of leisure screen time in particular have been associated with heightened psychological distress [8, 9]. High levels of sedentary behaviour can, however, exist alongside high levels of physical activity [10].

Research into the role of physical activity as a treatment for existing mental illness (such as anxiety and depression) as well as prevention of recurrence of illness, has a fairly extensive history although has predominantly focused on middle-aged and elderly people [11, 12]. The role of physical activity in enhancing mental health and preventing mental illness has, however, only relatively recently received attention. Results among adolescents are, however, inconsistent and the methodological quality of many studies has been questioned [7]. A recent cross-sectional study showed that low levels of physical activity were associated with increased prevalence of symptoms of depression and anxiety among Norwegian adolescents [13]. A further Norwegian cross-sectional study [9] documented an association between higher levels of physical activity, (> 11 h per week) and psychological distress among adolescents; however, this was not the case for middle and lower levels of physical activity. A European cross-sectional study found no evidence of the benefit of daily physical activity for mental health in adolescents [14]. Any relationship between physical activity and mental health, however, may not be linear. Rather, there may be an optimum range within which physical activity can protect against future mental illness.

Cross-sectional studies are especially limited in the extent to which the putative role of physical activity in prevention can be explored. Longitudinal studies can, in theory, better explore the temporal role of physical activity in this regard. However, they are currently few in number. One longitudinal study reported that low levels of physical activity were associated with symptoms of anxiety and depression among Norwegian adolescents, particularly among boys [13, 15]. However, no association was found between a decline in physical activity and a change in mental health over a 1-year period in a Dutch longitudinal study [16]. A recent longitudinal study among adults (40–60 years) reported that physical activity was associated with a reduced need for psychotropic medication [17]. Overall, research on physical activity and other factors associated with initiation of psychotropic drugs among adolescents is scarce.

To the best of our knowledge, there are few longitudinal studies that report on physical activity and its association with mental health in youth. Furthermore, there is a need for methodologically stronger studies that can contribute evidence to this field [18]. Thus, the purpose of this study was to investigate the association between physical activity and mental health in terms of subsequent use of psychotropic drugs (antidepressants and hypnotic use) in adolescents aged 15–16 years.

## Methods

### Data

**Fig. 1:**
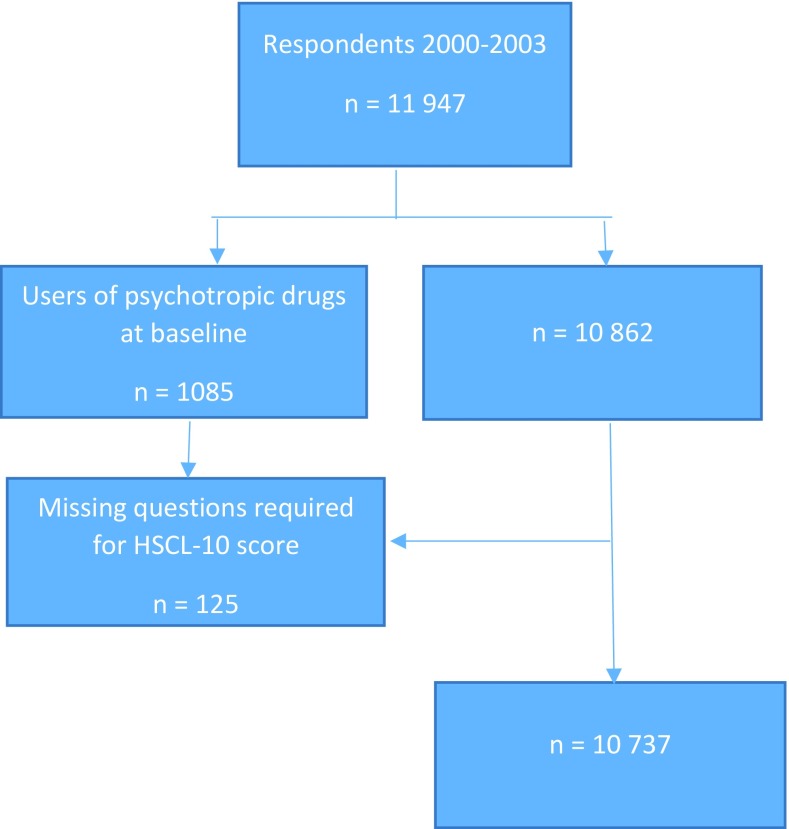
Flowchart for the study population: Norwegian Youth Health Survey in 2000–2003

This study is based on information retrieved from the Norwegian Youth Health Surveys, conducted by the Norwegian Institute of Public Health, and linked to the prescription data from the Norwegian Prescription Database (NorPD) [19]. A cross-sectional survey was conducted in the period 2001–2004 among 15–16 years in all secondary schools from 6 counties (Hedmark, Oppland, Troms, Finmark, Nordland, and Oslo) in Norway. The current study includes data from surveys conducted between 2000 and 2003 from 5 counties (-Nordland), covering both rural and urban regions. The adolescents completed a paper-and-pencil self-administered questionnaire at school during lesson time, and all students in grade 10 were invited to participate. To provide students with clarification on any matter related to the questionnaire, a researcher was present, and adolescents not present on the day of the data collection were asked to fill in the questionnaire later. In our analysis, 125 individuals were removed from the dataset due to non-response on the measure for psychological distress—a Hopkins Symptom Checklist-10 (HSCL-10) score. The participants who were psychotropic drug users at baseline (*n* = 1085) were excluded to study the incident use of psychotropic drugs. In total, the study population included 10,737 adolescents with a participation rate of 87%. Figure 1 shows the population in 2000–2003.

### Norwegian prescription database

Prescription data on psychotropic drug use in 2004–2013 were drawn from the NorPD, which covers the entire Norwegian population (approximately 5.2 million inhabitants). This is a valid and reliable data source for studying use of prescription drugs [19]. From January 2004, all pharmacies in Norway have been legally obliged to submit all electronic data on prescriptions to the Norwegian Institute of Public Health. The NorPD contains information on dispensed prescriptions to individual patients living outside institutions, whether or not reimbursed. Drugs administered to patients while in hospital are not reported to the NorPD. The drugs are classified according to the Anatomical Therapeutic Chemical (ATC) classification system [20]. The data in this study were patients’ unique personal identity number (encrypted), drug information [ATC code, the amount dispensed in defined daily doses (DDDs)], gender and age.

### The outcome measure

Incident psychotropic drug use was defined by the following ATC codes: N05C (hypnotics and sedatives), N06A (antidepressants), and R06AD01 (alimemazine). The systemic antihistamine alimemazine has long been used for childhood insomnia in Norway [21], and was, therefore, included as a hypnotic drug. Incident use was defined when an individual (non-user of a psychotropic drug at baseline) had a psychotropic drug dispensed at least once during the period between January 1, 2004 and December 31, 2013. The unique encrypted personal identity number was used to link the data from the Norwegian Youth Health Surveys and NorPD.

### Questionnaire and variable definitions

Data were extracted from the questionnaire, which included questions on health and lifestyle behaviour. The questions used in this paper relate to physical activity, psychological distress and screen-based sedentary behaviour, together with other lifestyle factors.

Table 1 shows the questions, the response alternatives, and the variable definitions used in the current study.

**Table 1:** The Norwegian Youth Health Survey 2000–2003: questions, response alternatives and variable definitions

Questions	Response alternatives	Variable definitions
*Psychological distress. The Hopkins Symptom Checklist-10 (HSCL-10)*		
Have you been troubled by any of these in the course of the past week (including today)? Sudden feeling of fear for no reason, feel afraid or anxious, feel faint or dizzy, feel tense or harassed, fell guilty (easily blame yourself), sleepless, feel burden, feeling of hopelessness for the future	Not at all (1) A little (2) Quite a bit (3) Extremely (4)	Psychological distress: HSCL score ≥ 1.85
*Physical activity*		
About how many hours peer week do you spend on physical activity?	Up to 1 h (1), 1–2 h (2), 3–4 h (3), 5–7 h (4), 8–10 h (5), more than 11 h (6)	< 5 h = Low activity group (0) > 5 h = High activity group (1)
*Sedentary behaviour*		
Outside school hours: How many hours per school day (Monday–Friday) do you sit, on average, in front of a TV, video and/or PC (games and internet)?	Up to 1 h (1), 1–2 h (2), 3–5 h (3), 6 h + (4)	< 2 h = not sedentary (0) > 2 h = sedentary (1)
*Self-perceived health*		
What is your current health status?	Poor (1), Not so good (2), Good (3), Very good (4)	Poor (0), Good (1)
*Use of medicines*		
How often in the course of the last 4 weeks has you taken the following medicines? Sedatives, Transquillisers, Anti-depressives	Daily (1), Every week, but not daily (2), Less often than every week (3), Not taken during the last weeks (4)	
*Smoking*		
Do you smoke, or have you smoked earlier?	No and never (1), Yes, but I have stopped (2), Yes, at times (3), Yes and daily (4)	Nonsmokers (0), Smoker (1)
*Alcohol*		
Have you ever drunk so much alcohol that you get drunk?	No and never (1), Yes, once (2), Yes, 2–3 times (3), Yes 4–10 times (4), Yes, 10 times (5)	Non consumer (0), Light consumer (1), Moderate consumer (2)
*Educations plans*		
University/college; upper secondary school; vocational education (upper secondary school); Not decided; other	What is the highest education considered? One alternative	Higher education (1), High School (2), Vocational education (3), Indefinite (4), Others (5)
*Family income*		
I think that our family, in relation to other families in Norway, has the following:	Poor economics (1), Moderate economics (2), Good economics (3), Very good economics (4)	Poor income (0), Moderate income (1) Good income (2)

### Measurement of leisure-time physical activity

Participants were asked how many hours per week they spent on physical activity that make them sweat and/or out of breath outside of school (Monday–Sunday), categorized as: 0, 1–2, 3–4, 5–7, 8–10, or 11 h or more per week. The term ‘physical activity’ included various types of activities in leisure time, both organized and unorganized.

### Measurement of psychological distress

Psychological distress at baseline was measured using Hopkins Symptom Checklist (HSCL-10). The HSCL-10 is derived from the widely used HSCL-25 [22] and captures symptoms of nervousness and depression during the previous week. This screening instrument has been validated and reported to have the potential to measure psychological distress among adolescents [23, 24]. The 10 questions, response alternatives and variable definitions are shown in Table 1. Adolescents were given no mean score and were excluded from the study if answering fewer than 6 of the 10 questions (Fig. 1). Missing values for the adolescents answering on 7, 8 and 9 questions were replaced with the sample mean value for each item.

### Measurement of other variables

Sedentary behaviour was based on self-report on the following question: outside school, how many hours per school day (Monday–Friday) do you sit, on average, in front of a TV, video and/or PC (games and Internet)? Responses were categorized as: up to 1, 1–2, 3–5 or 6 h or more. Self-reported general health, smoking, alcohol use, education plans and family income were also included (Table 1).

## Analysis

All analyses were carried out using SPSS 20.0 for Windows. The study population was stratified according to physical activity and gender. Baseline characteristics were presented as proportions with 95% confidence intervals (CI) in each stratum. No overlap of the CI was considered significant at the 5% level. The participants were followed prospectively by linkage on an individual level to prescription data on psychotropic drugs from the NorPD (antidepressant and hypnotic drug use from 2004 and onwards). Incident use was calculated by the number of new hypnotic or antidepressant users during a period (2004–2007, 2008–2010, 2011–2013), divided by the size of population at the start of the period minus the previous incident users.

Multinomial logistic regression analyses were used to examine the extent to which physical activity was associated with incident use of antidepressants and hypnotics, adjusted for confounding variables. A *p* value of ≤ 0.05 was set as the level for statistical significance. Associations were presented as odds ratios (OR) with 95% CI. To investigate different time intervals three models were tested, stratified into different periods: 2004–2007, 2008–2010 and 2011–2013. This was done for both antidepressants (N06A) and hypnotics (N05C and R06AD01) separately. Associations were adjusted for self-reported psychological distress, screen-based sedentary behaviour, family income, education plans and gender.

Interaction analysis was used to examine the influence of psychological distress and gender on the strength of the relationship between physical activity and incident use of psychotropic drugs (antidepressant and hypnotic use). This was done separately for incident antidepressant use and hypnotic use. Possible interaction effects were examined using the Likelihood Ratio test (LR test), contrasting models with and without interaction terms. The main effect model included physical activity, psychological distress, screen-based sedentary behaviour, family income, education plans and gender as independent variables, and was tested against models including interactions between physical activity by psychological distress and physical activity by gender. The incremental change in log-likelihood between the main effect models and models including interactions was not significant. Thus, the fit was not improved with the other interaction models. Therefore, only the main effect model is presented in the results.

## Results

Table 2 shows the baseline characteristics for adolescents who were non-users of psychotropic drugs when surveyed in 2000–2003.

**Table 2:** The Norwegian Youth Health Surveys: baseline characteristics for adolescents aged 15–16 years who were non-users of psychotropic drugs when surveyed in 2000–2003

	Total (*N* = 10,862)		Boys (*N* = 5471)		Girls (*N* = 5391)	
	High activity group^a^ (*N* = 4246) *n* (%; 95% CI)	Low activity group^b^ (*N* = 6329) *n* (%; 95% CI)	High activity group^a^ (*N* = 2694) *n* (%; 95% CI)	Low activity group^b^ (*N* = 2667) *n* (%; 95% CI)	High activity group^a^ (*N* = 1552) *n* (%; 95% CI)	Low activity group^b^ (*N* = 3663) *n* (%; 95% CI)
Psychological distress (HSCL score ≥ 1.85)	459 (10.8; 9.9–11.7)	1116 (17.6; 16.7–18.6)	158 (5.9; 5.0–6.8)	234 (8.8; 7.7–9.9)	301 (19.4; 17.4–21.4)	882 (24.1; 22.7–25.5)
*Sedentary behaviour, screen time after school*						
< 1 h	541 (12.8; 11.8–13.9)	691 (11.0; 10.2–11.8)	294 (11.0; 9.8–12.2)	244 (9.2; 8.1–10.3)	247 (16.1; 14.2–17.9)	447 (12.3; 11.3–13.4)
1–2 h	1444 (34.3; 32.8–35.7)	1997 (31.8; 30.6–32.9)	864 (32.3; 30.5–34.1)	741 (27.9; 26.2–29.6)	580 (37.7; 35.3–40.1)	1256 (34.6; 33.1–36.2)
3–5 h	1496 (35.5; 34.1–37.0)	2480 (39.5; 38.3–40.7)	971 (36.3; 34.5–38.1)	1062 (40.0; 38.2–41.9)	525 (34.1; 31.8–36.5)	1418 (39.1; 37.5–40.7)
> 5 h	733 (17.4; 16.3–18.5)	1114 (17.7; 16.8–18.7)	547 (20.4; 18.9–22.0)	606 (22.8; 21.2–24.4)	186 (12.1; 10.5–13.7)	508 (14.0; 12.9–15.1)
Poor self-perceived health	249 (5.9; 5.2–6.6)	879 (13.9; 13.0–14.7)	140 (5.2; 4.4–6.0)	332 (12.4; 11.2–13.7)	109 (7.0; 5.8–8.3)	547 (14.9; 13.8–16.1)
Smoker	1020 (24.0; 22.7–25.3)	2030 (32.1; 30.9–33.2)	595 (22.1; 20.5–23.7)	782 (29.3; 27.6–31.1)	425 (27.4; 25.2–29.6)	1248 (34.1; 32.5–35.6)
*Alcohol*						
Nonconsumer	730 (17.2; 16.1–18.3)	1329 (21.0; 20.0–22.9)	505 (18.7; 17.3–20.2)	569 (21.3; 19.8–22.9)	225 (14.5; 12.8–16.3)	760 (20.7; 19.4–22.1)
Light consumer	2131 (50.2; 48.7–51.7)	2974 (47.0; 45.8–48.2)	1317 (48.9; 47.0–50.8)	1276 (47.8; 46.0–49.7)	814 (52.4; 50.0–54.9)	1698 (46.4; 44.7–48.0)
Moderate consumer	1345 (31.7; 30.3–33.1)	1971 (31.1; 30.0–32.3)	844 (31.3; 29.6–33.1)	791 (29.7; 27.9–31.4)	501 (32.3; 30.0–34.6)	1180 (32.2; 30.7–33.7)
*Family income*						
Poor income	90 (2.1; 1.7–2.6)	231 (3.6; 3.2–4.1)	57 (2.1; 1.6–2.7)	100 (3.7; 3.0–4.5)	33 (2.1; 1.4–2.8)	131 (3.6; 3.0–4.2)
Moderate income	1259 (29.7; 28.3–31.0)	2125 (33.6; 32.4–34.7)	783 (29.1; 27.4–30.8)	820 (30.7; 29.0–32.5)	476 (30.7; 28.4–33.0)	1305 (35.6; 34.1–37.2)
Good income	2840 (67.0; 65.5–68.3)	3866 (61.1; 59.9–62.3)	1818 (67.5; 65.7–69.3)	1694 (63.5; 61.7–65.3)	1022 (65.9; 63.5–68.2)	2172 (59.3; 57.7–60.9)
*Education plans*						
University/college	2263 (53.3; 51.8–54.8)	2903 (45.9; 44.6–47.1)	1343 (49.9; 48.0–51.7)	1094 (41.0; 39.2–42.9)	920 (59.3; 56.8–61.7)	1809 (49.4; 47.8–51.0)
Upper secundary school	229 (5.4; 4.7–6.1)	370 (5.8; 5.3–6.4)	163 (6.1; 5.2–7.0)	161 (6.0; 5.1–6.9)	66 (4.3; 3.3–5.3)	209 (5.7; 5.0–6.5)
Vocational education	868 (20.4; 19.2–21.7)	1809 (28.6; 27.5–29.7)	678 (25.2; 23.5–26.8)	922 (34.6; 32.8–36.4)	190 (12.2; 10.6–13.9)	887 (24.2; 22.8–25.6)
Not decided	666 (15.7; 14.6–16.8)	932 (14.7; 13.9–15.6)	371 (13.8; 12.5–15.1)	327 (12.3; 11.0–13.5)	295 (19.0; 17.1–21.0)	605 (16.5; 15.3–17.7)
Other	161 (3.8; 3.2–4.4)	219 (3.5; 3.0–3.9)	99 (3.7; 3.0–4.4)	107 (4.0; 3.3–4.8)	62 (4.0; 3.0–5.0)	112 (3.1; 2.5–3.6)

^a^High activity group: ≥ 5 h per week

^b^Low activity group: < 5 h per week

### Physical activity

Table 2 shows baseline characteristics of the study population according to physical activity and gender. Overall, a higher proportion of the girls (3663/5391, 67.9%; 95% CI 66.7–69.2) were in the low activity group (< 5 h per week in leisure time) compared with the boys (2667/5471, 48.7%; 95% CI 47.4–50.1).

### Health and lifestyle habits

Overall, and in gender subgroups, adolescents in the low activity group reported significantly poorer psychological distress, poorer general health and more smoking compared with the rest of the study population. In total, 24.1% of the girls and 8.8% of the boys in the low activity group reported psychological distress. Irrespective of physical activity, a greater proportion of the girls more frequently reported psychological distress.

### Family income and education plans

Regarding education plans, 53.3% of the adolescents in the high activity group (≥ 5 h) reported university/college plans, compared to the 45.9% in the low activity group. In total 67% of the adolescents in the high activity group and 61.1% in the low activity group reported good family income.

Table 3 presents incident use of antidepressant and hypnotic drugs (2004–2013) in adolescents who were non-users of psychotropic drugs when surveyed in 2000–2003. Physical activity is stratified into high activity (≥ 5 h per week in leisure time) and low activity groups (< 5 h per week in leisure time).

**Table 3:** Incident use of antidepressants and hypnotics (2004–2013) in physical activity subgroups for adolescents surveyed in 2000–2003

	Total (*N* = 10,465)		Boys (*N* = 5289)		Girls (*N* = 5391)	
	High activity group^a^ (*N* = 4217) *n* (%; 95% CI)	Low activity group^b^ (*N* = 6248) *n* (%; 95% CI)	High activity group^a^ (*N* = 2668) *n* (%; 95% CI)	Low activity group^b^ (*N* = 2661) *n* (%; 95% CI)	High activity group^a^ (*N* = 1549) *n* (%; 95% CI)	Low activity group^b^ (*N* = 3627) *n* (%; 95% CI)
*Hypnotic* ^c^						
2004–2007	176 (4.2; 3.6–4.8)	334 (5.3; 4.8–5.9)	100 (3.7; 3.0–4.5)	108 (4.1: 3.3–4.8)	76 (4.9; 3.8–6.0)	226 (6.2; 5.4–7.0)
2008–2010	188 (4.7; 4.0–5.3)	312 (5.3; 4.7–5.9)	109 (4.2; 3.5–5.0)	116 (4.5; 3.7–5.4)	79 (5.4; 4.2–6.5)	196 (5.8; 5.0–6.6)
2011–2013	158(4.1; 3.5–4.7)	278 (5.0; 4.4–5.5)	92 (3.7; 3.0–4.5)	103 (4.2; 3.4–5.0)	66 (4.7; 3.6–5.9)	175 (5.5; 4.7–6.3)
2004–2013	522 (12.4; 11.4–13.4)	924 (14.8; 13.9–15.7)	301 (11.3; 10.1–12.5)	327 (12.3; 11.0–13.5)	221 (14.3; 12.5–16.0)	597 (16.5; 15.3–17.7)
*Antidepressants*						
2004–2007	163 (3.9; 3.3–4.5)	351 (5.6; 5.1–6.2)	83 (3.1; 2.5–3.8)	97 (3.6; 2.9–4.4)	80 (5.2; 4.1–6.3)	254 (7.0; 6.2–7.8)
2008–2010	136 (3.4; 2.8–3.9)	134 (2.3; 1.9–2.7)	81 (3.1; 2.5–3.8)	89 (3.5; 2.8–4.2)	53 (3.6; 2.7–4.6)	174 (5.2; 4.4–5.9)
2011–2013	109 (2.8; 2.3–3.3)	258 (4.5; 3.9–5.0)	57 (2.3; 1.7–2.9)	76 (3.1; 2.4–3.8)	53 (3.7; 2.8–4.7)	182 (5.7; 4.9–6.5)
2004–2013	406 (9.6; 8.7–10.5)	872 (14.0; 13.1–14.8)	221 (8.3; 7.2–9.3)	262 (9.8; 8.7–11.0)	185 (11.9; 10.3–13.6)	610 (16.8; 15.6–18.0)
*Either Hypnotics OR antidepressants*						
2004–2007	215 (5.1; 4.4–5.8)	434 (6.9; 6.3–7.6)	123 (4.6; 3.8–5.4)	128 (4.8; 4.00–5.6)	92 (5.9; 4.8–7.1)	306 (8.4; 7.5–9.3)
2008–2010	259 (6.5; 5.7–7.2)	438 (7.5; 6.9–8.2)	151 (5.9; 5.00–6.9)	156 (6.2; 5.2–7.1)	108 (7.4; 6.1–8.8)	282 (8.5; 7.5–9.4)
2011–2013	204 (5.5; 4.7–6.2)	385 (7.2; 6.5–7.9)	114 (4.8; 3.9–5.6)	130 (5.5; 4.6–6.4)	90 (6.7: 5.3–8.0)	255 (8.4; 7.4–9.4)
2004–2013	742 (17.6; 16.5–18.7)	1395 (22.3; 21.3–23.4)	417 (15.6; 14.3–17.0)	456 (17.1; 15.7–18.6)	325 (21.0; 19.0–23.0)	939 (25.5; 24.5–27.3)

^a^High activity group: ≥ 5 h per week after school

^b^Low activity group: < 5 h per week after school

^c^Hypnotics is depend as either N05C or R06AD01

Overall and in gender subgroups, incident psychotropic drug use 1–13 years after participation in the Youth Health Surveys was higher among those who reported low activity (Table 3). The highest incidence of psychotropic drug use was observed among girls who reported low activity at baseline, of whom 25.5% had retrieved at least one psychotropic drug prescription, compared with 21.0% of remaining girls. Corresponding proportions of such use among boys were 17.1% among the low activity group compared with 15.6% among the remaining boys.

We observed a larger difference in incident use of antidepressants between adolescents in the high activity (9.6%) and low activity group (14.0%), compared to incident use of hypnotics (≥ 5 h: 12.4%, < 5 h: 14.8%). Incident antidepressant use was higher among girls (16.8%) compared to boys (9.8%) in the low activity group.

During the years 2004–2013 (2004–2007, 2008–2010 and 2011–2013), a weak decrease was observed in incident use of antidepressants between the 3-year time periods in the high activity group. Among girls, a significantly higher incidence of antidepressant use in those who reported low activity was the major contributor to an overall higher incidence in psychotropic drug use in this group.

Table 4 presents the association between physical activity, psychological distress, other factors and incident use of hypnotics.

**Table 4:** Multinominal logistic regression analysis of incident hypnotic^a^ use (2004–2013) in relation to combinations of physical activity and psychological distress (Youth Health Survey 2000–2003)

Variables	Model A			Model B			Model C		
	2004–2007	2008–2010	2011–2013	2004–2007	2008–2010	2011–2013	2004–2007	2008–2010	2011–2013
*None user, hypnotics*	OR (95% CI)	OR (95% CI)	OR (95% CI)	OR (95% CI)	OR (95% CI)	OR (95% CI)	OR (95% CI)	OR (95% CI)	OR (95% CI)
*Physical activity*									
< 1 h	1 (ref)	1 (ref)	1 (ref)	1 (ref)	1 (ref)	1 (ref)	1 (ref)	1 (ref)	1 (ref)
1–2 h	0.64 (0.48–0.85)	1.07 (0.78–1.48)	0.91 (0.65–1.28)	0.67 (0.50–0.89)	1.10 (0.79–1.51)	0.93 (0.66–1.30)	0.71 (0.53–0.96)	1.13 (0.81–1.58)	0.99 (0.69–1.41)
3–4 h	0.58 (0.43–0.77)	0.85 (0.61–1.18)	0.92 (0.66–1.30)	0.62 (0.46–0.83)	0.88 (0.63–1.23)	0.95 (0.68–1.34)	0.69 (0.51–0.93)	0.94 (0.67–1.33)	1.01 (0.71–1.44)
5–7 h	0.48 (0.35–0.65)	0.77 (0.55–1.09)	0.83 (0.58–1.18)	0.54 (0.39–0.74)	0.82 (0.58–1.16)	0.88 (0.61–1.25)	0.65 (0.47–0.89)	0.91 (0.64–1.31)	0.97 (0.67–1.41)
8–10 h	0.49 (0.34–0.71)	0.82 (0.56–1.21)	0.68 (0.45–1.04)	0.57 (0.40–0.83)	0.89 (0.60–1.32)	0.73 (0.48–1.12)	0.72 (0.49–1.05)	1.00 (0.66–1.50)	0.79 (0.50–1.24)
11 > h	0.63 (0.43–0.94)	1.07 (0.71–1.61)	0.68 (0.42–1.11)	0.72 (0.49–1.07)	1.15 (0.76–1.74)	0.72 (0.45–1.18)	0.95 (0.63–1.43)	1.34 (0.87–2.06)	0.83 (0.50–1.38)
*Psychological distress*									
HSCL score ≤ 1.84				1 (ref)	1 (ref)	1 (ref)	1 (ref)	1 (ref)	1 (ref)
HSCL score ≥ 1.85				2.61 (2.13–3.19)	1.82 (1.46–2.26)	1.65 (1.29–2.09)	2.30 (1.86–2.85)	1.63 (1.29–2.06)	1.57 (1.22–2.02)
*SB (screentime)*									
SB ≤ 2 h							1 (ref)	1 (ref)	1 (ref)
SB > 2 h							1.03 (0.85–1.24)	0.88 (0.73–1.07)	0.90 (0.74–1.10)
*Family income*									
Good income							1 (ref)	1 (ref)	1 (ref)
Moderate income							1.51 (1.25–1.83)	1.01 (0.82–1.23)	0.96 (0.77–1.19)
Poor income							2.17 (1.44–3.25)	1.72 (1.11–2.67)	1.32 (0.79–2.20)
*Education plans*									
Higher education							1 (ref)	1 (ref)	1 (ref)
High school							1.28 (0.85–1.91)	0.96 (0.62–1.49)	1.20 (0.79–1.83)
Vocational education							1.53 (1.23–1.91)	1.28 (1.03–1.61)	1.12 (0.99–1.42)
Other							0.98 (0.76–1.27)	1.09 (0.85–1.39)	1.00 (0.76–1.30)
*Gender*									
Boy							1 (ref)	1 (ref)	1 (ref)
Girl							1.42 (1.16–1.74)	1.27 (1.04–1.55)	1.24 (1.00–1.53)

The dependent variable consisted of four categories coded as years 2004–2007, years 2008–2010, years 2011–2013 and none users as the reference category.

^a^N05C or R06D01

We found associations between physical activity and incident hypnotic use for the 2004–2007 time period, but not for 2008–2010 and 2011–2013.

In the crude models for first time period 2004–2007, the odds of incident hypnotic use were lower for those who were physically active [OR: 0.64 (1–2 h), 95% CI 0.48–0.85, OR: 0.58 (3–4 h), 95% CI 0.43–0.47, OR: 0.48 (5–7 h), 95% CI 0.35–0.65, OR: 0.49 (8–10 h), 95% CI 0.34–0.71, OR: 0.63 (≥ 11 h), 95% CI 0.43–0.94)], compared to those who were physically inactive < 1 h. After adjustment for psychological distress, we observed a small change in OR, and only the association for ≥ 11 h became non-significant. After adjustment for other confounders, the association became non-significant for those who were physically active ≥ 8 h.

Psychological distress was significantly associated with incident use of hypnotics in all time periods (2004–2007, 2008–2010 and 2011–2013). The adolescents who reported poor family income (2004–2007: OR: 2.17 and 2008–2010: OR: 1.72), and vocational education (2004–2007: OR: 1.53 and 2008–2010: OR: 1.28), had a higher odds of being in the group of incident hypnotic users. Girls had higher odds for being an incident hypnotic user than boys after controlling for the other variables; however, none were significant in the last period (2011–2013).

Table 5 presents the association between physical activity, psychological distress, other factors and incident use of antidepressants.

**Table 5:** Multinominal logistic regression analysis of incident antidepressant use (2004–2013) in relation to combinations of physical activity and psychological distress (Youth Health survey 2000–2003)

Variables	Model A			Model B			Model F		
	2004–2007	2008–2010	2011–2013	2004–2007	2008–2010	2011–2013	2004–2007	2008–2010	2011–2013
*None user, hypnotics*	OR (95% CI)	OR (95% CI)	OR (95% CI)	OR (95% CI)	OR (95% CI)	OR (95% CI)	OR (95% CI)	OR (95% CI)	OR (95% CI)
*Physical activity*									
< 1 h	1 (ref)	1 (ref)	1 (ref)	1 (ref)	1 (ref)	1 (ref)	1 (ref)	1 (ref)	1 (ref)
1–2 h	0.71 (0.53–0.95)	0.66 (0.48–0.92)	0.77 (0.56–1.07)	0.74 (0.55–0.99)	0.68 (0.49–0.95)	0.79 (0.57–1.09)	0.80 (0.59–1.08)	0.75 (0.53–1.06)	0.88 (0.63–1.24)
3–4 h	0.69 (0.52–0.92)	0.67 (0.48–0.93)	0.58 (0.41–0.82)	0.75 (0.56–1.01)	0.71 (0.51–0.98)	0.61 (0.43–0.86)	0.83 (0.61–1.12)	0.80 (0.57–1.13)	0.72 (0.50–1.04)
5–7 h	0.46 (0.33–0.63)	0.52 (0.36–0.73)	0.37 (0.25–0.55)	0.52 (0.38–0.72)	0.56 (0.39–0.80)	0.40 (0.27–0.59)	0.63 (0.45–0.89)	0.66 (0.46–0.96)	0.51 (0.34–0.76)
8–10 h	0.49 (0.34–0.72)	0.40 (0.25–0.63)	0.54 (0.36–0.82)	0.59 (0.40–0.87)	0.45 (0.28–0.71)	0.59 (0.39–0.90)	0.76 (0.51–1.13)	0.52 (0.32–0.84)	0.79 (0.50–1.24)
11 > hours	0.59 (0.39–0.89)	0.72 (0.47–1.11)	0.46 (0.28–0.76)	0.69 (0.46–1.05)	0.79 (0.51–1.23)	0.50 (0.30–0.83)	0.90 (0.58–1.40)	1.02 (0.65–1.61)	0.76 (0.45–1.30)
*Psychological distress*									
HSCL score ≤ 1.84				1 (ref)	1 (ref)	1 (ref)	1 (ref)	1 (ref)	1 (ref)
HSCL score ≥ 1.85				3.24 (2.66–3.93)	2.30 (1.83–2.90)	1.95 (1.52–2.50)	2.61 (2.12–3.22)	1.92 (1.50–2.47)	1.75 (1.35–2.27)
*SB (screentime)*									
SB < 2 h							1 (ref)	1 (ref)	1 (ref)
SB > 2 h							1.09 (0.91–1.32)	0.81 (0.65–1.00)	0.95 (0.76–1.18)
*Family income*									
Good income							1 (ref)	1 (ref)	1 (ref)
Moderate income							1.34 (1.11–1.63)	1.34 (1.08–1.66)	1.15 (0.92–1.45)
Poor income							2.44 (1.67–3.58)	1.89 (1.17–3.05)	1.27 (0.72–2.23)
*Education plans*									
Higher education							1 (ref)	1 (ref)	1 (ref)
High school							1.25 (0.82–1.90)	1.44 (0.92–2.24)	1.01 (0.60–1.72)
Vocational education							1.71 (1.38–2.13)	1.42 (1.10–1.83)	1.65 (1.28–2.12)
Other							1.19 (0.93–1.53)	1.49 (1.14–1.94)	1.14 (0.85–1.53)
*Gender*									
Boy							1 (ref)	1 (ref)	1 (ref)
Girl							1.72 (1.40–2.11)	1.32 (1.06–1.66)	1.88 (1.48–2.39)

The dependent variable consisted of four categories coded as years 2004–2007, years 2008–2010, years 2011–2013 and none users as the reference category

In the crude model for the time periods 2004–2007, 2008–2010 and 2011–2013, the odds of incident antidepressant use were lower for physically active adolescents [2004–2007: OR 0.46–0.71, 2008–2010: OR 0.40–0.67 (non sig for ≥ 11 h) and 2011–2013: OR 0.37–0.58 (non sig for 1–2 h)], compared to those who were physically inactive < 1 h. After adjustment for psychological distress, we observed only a small change in OR. However, after controlling for psychological distress, screen-based sedentary behaviour, family income, education plans and gender, the association became non-significant in all time periods (2004–2007, 2008–2010 and 2011–2013) except in those who were physically active for 5–7 and 8–10 h in the period 2008–2010.

Psychological distress was significantly associated with incident antidepressant use in all periods (2004–2007, 2008–2010 and 2011–2013). The adolescents who reported poor family income (2004–2007: OR = 2.44, 2008–2010: OR = 1.89, 2011–2013: OR = 1.27) and vocational education (2004–2007: OR = 1.71, 2008–2010: OR = 1.42, 2011–2013: OR = 1.65), had a higher risk of being in the group of incident hypnotic users. However poor family income was not significant in the period 2011–2013. Girls had higher odds of being an incident antidepressant user than boys after controlling for the other variables.

## Discussion

Our study shows that physical activity is associated with incident hypnotic use for the 2004–2007 time period, but not for the 2008–2010 and 2011–2013 time periods. A weak association was found between physical activity and antidepressants in the crude model, while most odds ratios were non-significant in the adjusted model controlling for socioeconomic and other factors. Given the results, physical activity seems to be a stronger predictor for incident use of hypnotics than incident use of antidepressants in the first period. As a whole, these findings suggest that incident use of hypnotics and antidepressants over the 13-year period are mainly explained by variables other than physical activity. Our results showing discrepant patterns depending on the outcome measures is in agreement with other studies among adolescents [9, 25]. We are, however, not aware of any longitudinal studies among adolescents that have looked at the association between physical activity and incident use of psychotropic drugs. Our results are inconsistent with parallel studies among adults. A Danish study among adults reported that physical activity was associated with a reduced need for psychotropic medication [17]. Furthermore, the study reported that high doses of psychotropic medication use were associated with less physical activity. Another prospective cohort study among adults reported that leisure-time physical activity was associated with decreased risk of psychotropic medication [26].

While there are few, if any, adolescent studies on physical activity and its association with psychotropic drug use, there are a number of studies on the association between physical activity and mental health. These studies are relevant, because they highlighted the possible role of physical activity in relation to mental health. The results are, however, ambiguous. A meta-analysis reported overall, a small but significant effect of physical activity on mental health in children and adolescents aged 3–18 [27]. A prospective study among Dutch adolescent (age period 13–18), found no evidence that physical activity (frequency, duration or intensity) protect against the development of depressive episodes neither among girls nor boys [28]. This is in line with another Dutch study, which reported no significant association between a decline in physical activity (over a 1-year period) and a change in mental health among adolescents (mean age 13.6 years) [16]. McPhie and Rawana [29], showed in a longitudinal study that adolescents who reported higher frequencies of physical activity were more resilient to developing depressive symptoms. A further longitudinal study reported that low levels of physical activity in adolescents aged 14/15 were associated with poor mental health 7 years later in girls, but not in boys [18].

We did not observe any interaction effects on physical activity and psychological distress or on physical activity and gender, that is to say, the strength of the association between hypnotics and antidepressants and physical activity was unaffected by psychological distress and gender. This is in line with a study among Dutch adolescents, in which the results showed no interaction between physical activity and gender [28].

The methodological quality of many studies on adolescents has been questioned, particularly in relation to the varying measurements of mental health and physical activity in different studies and the neglect of factors such as psychological climate and social interactions [7]. The weak association between physical activity and mental health (measured by incident use of hypnotics and antidepressants) found in this study might, be due to—at least in part— the methods used, in particular to lack of information about physical activity at the time the psychotropic drugs was prescribed. The weak association between physical activity and mental health may also be due to the measurement of physical activity and mental health as well as not  accounting for different forms of physical activity and the social context in which they take place.

The present study showed that in 2000–2003 girls were less active than boys. This finding is in line with other international studies of activity levels in European adolescents aged 15 [14, 30]. Overall, the adolescents who had a low activity reported psychological distress, poorer general health, and more smoking compared with the rest of the study population. This is in line with a study among 15–16-year-old adolescents in Iceland, with girls being less active and reporting higher levels of depressive symptoms than boys [31].

Psychological distress in the current study showed an independent effect on incident hypnotic and antidepressant use, which is in line with other studies [32]. This was expected, that is to say, psychological distress is an indicator for psychotropic drug use in the first place. Initiation rates were highest among girls, who reported psychological distress and low physical activity, of whom 24.1% (antidepressant) and 23% (hypnotics) had started drug therapy before the age of 29. We observed a higher initiation rate among girls compared with boys, irrespective of physical activity.

Irrespective of physical activity level at baseline, incident antidepressant and hypnotic use in the current study was higher for girls compared to boys. This is in line with other Norwegian studies; Hartz et al. [4] reported that girls used more psychotropic drugs than boys during adolescence for antidepressants and hypnotics. Antidepressants use has increased, especially among girls (15–17 years), and is used twice as much among adolescent girls than among boys [4]. Furthermore, there is a trend among Norwegian adolescents of increasing hypnotic drug use, illustrated by a threefold increase in hypnotic drug use among females from 13 to 17 years of age [33]. The increase in hypnotic drug use in adolescents may be explained by an increasing prevalence of sleeping problems. Antidepressant use has increased markedly from 2005 to 2012 in five western countries, and the increase differed across years and countries, i.e., the increase was greatest in 15–19 years in Denmark, Germany and the United States, and in 10–14 years in the Netherlands and the United Kingdom [34].

### Strengths and limitations

A strength of this study is it large sample size, which provides sufficient power to detect relevant differences and reduces the risk of type II errors. The longitudinal design, with a follow-up period of 13 years, made it possible to investigate prospectively the association between physical activity and the use of psychotropic drugs in adulthood ‘in a more proper way than is possible in cross-sectional studies’. The NorPD is a complete resource for assessing prescription drug use in large populations and with the potential for long-term follow-up [35]. However, we do not know whether the dispensed drugs registered in our study reflect actual drug use, and we have no information about drugs used among adolescents in hospital. Furthermore, we have no way of knowing if (the incident) prescribing of psychotropic drugs to adolescents over the follow-up period changed in any way. Self-reported measures of sedentary behaviour (from Monday to Friday) and physical activity (after school and at weekends) were used, which might lead to misclassification or measurement error. Indeed, incident use of psychotropic drugs may not only be an outcome but may also act as an exposure in leading to physical inactivity and/or sedentary behaviour. Our focus on leisure time may have led to an under-estimation of physical activity and sedentary behaviour, because it does not take account of school-based activity nor, in the case of sedentary behaviour, time at the weekend. A limitation with the study is that data on physical activity were collected only at one point in time. This weakness is addressed by analysing the associations for different time periods.

## Conclusions

Physical activity does not indicate any association with later use of antidepressants, and the significant association with incident hypnotic drug use was for short-term follow-up only and disappeared on longer term follow-up periods. Given the scarcity of longitudinal studies examining the association between physical activity and mental health as well as psychotropic drug use among young people, the current study adds to previous research.
